# Long Non-Coding RNAs: Role in Testicular Cancers

**DOI:** 10.3389/fonc.2021.605606

**Published:** 2021-03-09

**Authors:** Chiara Bresesti, Valeria Vezzoli, Biagio Cangiano, Marco Bonomi

**Affiliations:** ^1^ Department of Endocrine and Metabolic Medicine, IRCCS Istituto Auxologico Italiano, Milan, Italy; ^2^ Lab of Endocrine and Metabolic Researches, IRCCS Istituto Auxologico Italiano, Cusano Milanino, Italy; ^3^ Department of Medical Biotechnology and Translational Medicine, University of Milan, Milan, Italy

**Keywords:** testis cancer, testicular germ-cell tumors, seminomas, non-seminomas, hypogonadism

## Abstract

In the last few years lncRNAs have gained increasing attention among the scientific community, thanks to the discovery of their implication in many physio-pathological processes. In particular, their contribution to tumor initiation, progression, and response to treatment has attracted the interest of experts in the oncologic field for their potential clinical application. Testicular cancer is one of the tumors in which lncRNAs role is emerging. Said malignancies already have very effective treatments, which although lead to the development of quite serious treatment-related conditions, such as secondary tumors, infertility, and cardiovascular diseases. It is therefore important to study the impact of lncRNAs in the tumorigenesis of testicular cancer in order to learn how to exploit them in a clinical setting and to substitute more toxic treatments. Eventually, the use of lncRNAs as biomarkers, drug targets, or therapeutics for testicular cancer may represent a valid alternative to that of conventional tools, leading to a better management of this malignancy and its related conditions, and possibly even to the treatment of poor prognosis cases.

## Introduction to Long Non-Coding RNAs

### Toward lncRNAs Definition

Long non-coding RNAs (lncRNAs) is a relatively recent definition for a class of transcripts that has been studied for decades. Members of this class have been known since the early 1990s, when the lncRNA *XIST* was found to be covering the inactive X chromosome in both human and mice (1, 2), and the lncRNA *H19* was described as being implicated in the parental imprinting of the homonymous gene in mice (3).

However, a systematic classification of non-coding RNAs (ncRNAs) was not deemed necessary until the advent of large-scale analysis of transcriptomes, when the real relevance of this class of transcripts became apparent. Thanks to genomic projects such as FANTOM and ENCODE, we now know that 80% of the human genome is indeed transcribed, but only 2% of it is restricted to protein coding (4, 5).

**Figure 1:**
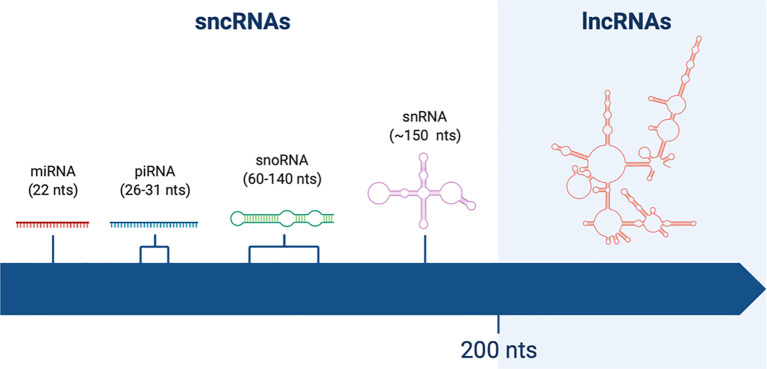
Schematic representation of non-coding RNAs (ncRNAs) of different lengths. Short non-coding RNAs (sncRNAs) are shorter than 200 nucleotides (nts). Among them one can find miRNAs (22 nts), piRNAs (26–31 nts), snoRNAs (60–140 nts), and snRNAs (~150 nts). On the other hand, long non-coding RNAs (lncRNAs) are longer than 200 nts, reaching up to thousands of nts of length.

The classification of ncRNAs was initially based on transcripts length: a cut-off value of 200 nucleotides (nts) was arbitrarily chosen, more on the basis of RNA binding to silica columns during purification rather than its functional meaning (6). Therefore, the term long non-coding RNAs was coined to identify RNA molecules longer that 200nt, in contrast to short non-coding RNAs (sncRNAs) (**Figure 1**).

SncRNAs have been fascinating the scientific community for the last couple of decades. In particular miRNAs, a class of 22 nts long ncRNAs involved in the process of RNA interference (RNAi), are now recognized as powerful and ubiquitarians regulators of gene expression at transcriptional and post-transcriptional level. These small regulatory RNAs pair with a target mRNA, causing its endonucleolytic cleavage or inhibiting its translation, ultimately leading to a reduction of its protein product (7).

Other members of the class of sncRNAs are small nuclear RNAs (snRNAs), which are part of the spliceosome complex, small nucleolar RNAs (snoRNAs), involved in RNA processing, and piwi-interacting RNAs (piRNAs), which as miRNAs are also able to trigger the process of RNAi to regulate gene expression (8).

On the other hand, lncRNAs class is gaining more and more interest among the scientific community, as new lncRNAs genes are discovered and characterized every day. In fact, the number of annotated lncRNA is quickly approaching that of protein-coding transcripts, as the current GENCODE Release (version 34) reports 17,960 and 19,959 genes respectively.

### Characteristics: Structure, Conservation, Expression

LncRNAs are relevant not only for their numerosity, but also for their multiple biological functions. They have been associated with a number of cellular and biological events, including gene regulation at transcriptional and post-transcriptional level, transcripts maturation and stability, organization of nuclear architecture, and regulation of interacting proteins and RNAs (9–11). Then, it is not surprising that the number of physiological and pathological processes in which lncRNAs are involved is increasing every day.

This wide range of functionalities can be partly explained by lncRNAs structural heterogeneity. Most of them possess features reminiscent of protein-coding genes, such as a 5’ cap, alternative splicing and the presence of a polyA tail. Some lncRNAs can even contain ORFs and have both protein-coding and coding-independent functions (12). However, many others lack such features, and are nevertheless active as primary transcripts (10, 13).

However, there are some structural characteristics that seem to be common to all lncRNAs, one of which is their enrichment in transposable elements (TEs). In fact, it has been estimated that 41% of lncRNA nucleotides derive from TEs and 83% of lncRNAs contain at least part of a TE (14).

Even though TEs have long been regarded as purely parasitic elements, evidence suggest their peculiar role in lncRNAs evolution and functionality. Specific TEs are strongly and non-randomly enriched or depleted from lncRNAs sequences, indicating that their presence there is under selective pressure (15). Moreover, different studies proved how TEs constitute lncRNA functional domains, with roles ranging from the regulation of target mRNA transcription, stability and translation, to the regulation of entire gene networks (16–19).

LncRNAs are relatively young genes, and their rapid evolution is reflected by their poor sequence conservation among tetrapods and thus their general lack of identifiable orthologs (20, 21). This can be partly explained by their enrichment in TEs, since the insertion loci or even the presence of some classes of TEs, like Alus, are restricted to higher organisms, usually primates.

However, their poor sequence conservation does not necessarily imply that lncRNAs function cannot be conserved in different species. In fact, many lncRNAs are part of syntenic loci, which suggests a functional relatedness in spite of a lack of sequence homology (21). Moreover, there are different examples of poorly conserved lncRNAs among different species exhibiting a conserved function, such as megamind/*TUNA*, which contributes to brain development in zebrafish, mouse, and human (22, 23). Therefore, it is possible that RNA molecules need less sequence conservation to maintain their function compared to proteins.

Opposed to what happens for lncRNA gene bodies, lncRNA promoters show a higher sequence conservation than protein-coding gene promoters (24). This fact not only supports lncRNAs recent emergence (promoters had a limited time frame to diverge), but also that the control of their expression, low but finely regulated, is particularly important (6, 25, 26). Furthermore, their expression is markedly tissue or lineage-specific, much more so than that of protein-coding genes (26, 27).

Possibly compensating for their low expression level, lncRNAs stability appears to be comparatively high, with a study showing that up to 70% of analyzed lncRNAs have half-lives longer than 2 h, and even longer than 12 h for 3% of analyzed lncRNAs (28).

### Classification and Functions

LncRNAs complexity in terms of structure and biological role poses a major challenge to their classification. Therefore, lncRNAs can be classified following different criteria, starting from their genomic localization to end with their mode of action or function.

Regarding lncRNAs genomic localization it is important to consider that their position is always defined with regard to protein-coding genes present in the same genomic region (29):

- Intronic lncRNAs originate from the introns of protein-coding genes
- Enhancer lncRNAs originate from enhancer regions of protein-coding genes
- Intergenic lncRNAs originate from the region between two protein-coding genes

LncRNAs that overlap broad portions of a protein-coding gene have also been described and, in this case, they are distinguished based on their orientation (30):

- Sense-overlapping lncRNAs originate from the same sense DNA strand
- Natural antisense transcripts (NATs) from the opposite sense DNA strand

Finally, there are bidirectional/divergent lncRNAs, whose transcription starts from the promoter of a protein-coding gene, but in the opposite direction. Given that transcriptional regulatory elements can initiate transcription bi-directionally (30–32), these lncRNAs were initially considered as by-products of a leaky transcriptional activity. However, some bidirectional lncRNAs have been found to have a function, indicating that the biological relevance of this class of transcripts deserves more attention (33).

**Figure 2:**
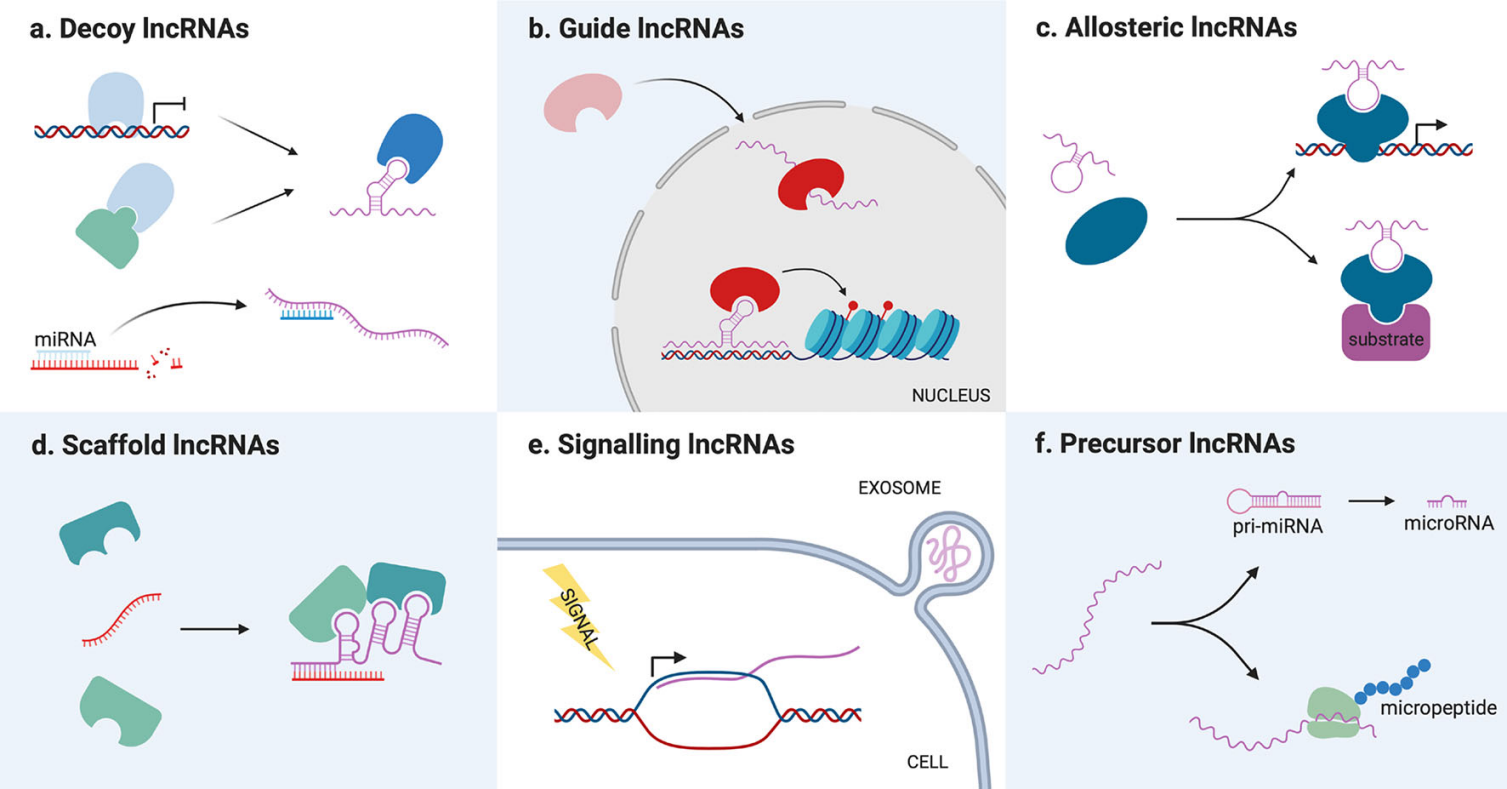
Schematic representation of long non-coding RNAs (lncRNAs) different molecular mechanisms. **(A)** Decoy lncRNAs titrate away proteins or miRNAs from their molecular partners, inhibiting their function. **(B)** Guide lncRNAs bind protein partners and direct them toward a specific cellular compartment or genomic target. **(C)** Allosteric lncRNAs interact with transcription factors or enzymes causing structural modifications that modify their activity. **(D)** Scaffold lncRNAs bind different molecular partners (proteins or RNAs) allowing them to interact or assemble into a complex. **(E)** Signaling lncRNAs are transcribed following a stimulus for which they act as signal molecules. They can be packed in exosomes and transmitted to other cells. **(F)** Precursor lncRNAs are processed into miRNAs or translated into functional micropeptides.

As previously mentioned, lncRNAs can also be classified on the basis of their mode of action (**Figure 2**). In general, lncRNAs functionality resides in their capacity to bind and regulate a molecular partner (protein, RNA or genomic DNA). This interaction is possible either *via* base-pair interactions or through their secondary structure, which is complex enough to mediate the interaction with proteins. Once they have bound their partners, lncRNAs can regulate them in different ways, acting as decoys, guides, scaffolds for the assembly of riboproteic complexes or allosteric modulators.

Decoy lncRNAs acts as “molecular sinks” for different target molecules, binding, and titrating them away to inhibit their function (34). Such target molecules can be chromatin modifiers or other nuclear factors, thereby their titration alters DNA transcription (34), but also cytoplasmatic proteins or other ribonucleic molecules. Among this class of lncRNAs, the so-called miRNA sponges have recently gained great interest among the scientific community, with dozens of new examples that appeared in the last couple of years. These lncRNAs are able to sequester miRNA through base-pairing interactions, preventing them from binding their usual target mRNAs and therefore upregulating the corresponding proteins (35).

One of the best characterized miRNA sponge is metastasis-associated lung adenocarcinoma transcript 1 (MALAT1). In the last few years, MALAT1 function has been linked to its capacity to sponge dozens of miRNAs in various conditions, including miR-133 in ischemia-reperfusion injuries (36), miR-150-5p in osteoarthritis (37) and several miRNas in different cancers (38, 39).

Guide lncRNAs are able to bind a protein partner and then direct its localization to a specific cellular compartment. This can mean both relocating a protein from the cytoplasm to the nucleus or guiding nuclear factors toward specific genomic regions. Modifying transcription factors location, lncRNAs can determine changes in gene expression recruiting chromatin modifiers or transcription factors in *cis* (on neighboring genes) or in *trans* (on distantly located genes), causing either an upregulation or gene silencing (6). For example, LincRNA-p21 acts as a guide *in trans* regulating the localization of heterogeneous nuclear ribonucleoprotein K (hnRNP-K), which is involved in mRNA processing and transport (40).

Scaffold lncRNAs serve as central platforms upon which different molecular components are assembled. Through this mechanism, lncRNAs can promote the interaction of an enzyme and its substrate, the assembly of (ribo)proteic complexes, and even the formation of bigger structures, such as nuclear bodies (14). For example, the lncRNA *NEAT1* is necessary for the formation and maintenance of paraspeckles, a nuclear compartment devoted to RNA transcription and processing, since it binds and direct the localization of many proteins that compose these structures (41–43).

LncRNAs can also act as allosteric modulators of enzymes or transcription factors. Binding their protein partner, such lncRNAs can cause conformational alterations that increase the enzyme catalytic activity, or the transcription factor ability to bind its genomic target. The main difference between this class of transcripts and guide/scaffold RNAs is that they only bind their protein partner, without interacting with the substrate of the enzyme or the genomic target of the transcription factor. One example is the lncRNA *ACOD1*, which binding to the enzyme glutamic-oxaloacetic transaminase (GOT2) near its substrate niche was found to increase its catalytic activity (44).

However, lncRNAs function can also be independent from that of interacting partners. In fact, lncRNAs can act as signal molecules for intercellular communication since they are often released in extracellular fluids, especially packed in exosomes (45). These vesicles provide a platform for cell-to-cell communication, allowing the exchange of different kinds of signal molecules between a donor and a recipient cell. In particular, exosome-derived lncRNAs have been described to favor tumorigenesis inducing a malignant phenotype in the recipient cell or the plastic modification of cells in the tumoral microenvironment (46).

The idea that lncRNAs can act as signal molecules makes sense from a biological perspective, since the expression of single lncRNAs occurs at very specific moments and locations to integrate developmental cues, interpret cellular context or react to a variety of stimuli. Moreover, the fact that lncRNAs are rarely transcribed provides a quick response, making them a kinetically sensible signal molecule.

Lastly, lncRNAs functionality can also reside in their ability to serve as a precursor for the synthesis of other molecules, which in turn determine a cellular event. As previously mentioned, lncRNA can code for micropeptides, which can play roles in physiological (47–52) and pathological settings (53, 54). The coding potential of lncRNAs is relatively little studied, but it is particularly interesting because it opens the possibility of the existence of protein-coding transcripts bearing also non-coding functions. On the other hand, lncRNA can also be processed into miRNA, constituting a substrate for their biosynthesis. Different lncRNAs have been described as miRNA reservoirs, but the most famous is *H19*, which is a precursor of the well-known miR-675 (55).

## Long Non-Coding RNAs Biological Roles

### Physiological Role and Pathogenic Potential

As illustrated above, lncRNAs are involved in a vast number of molecular events, regulating almost every aspect of gene expression, but also the function and localization of a number of proteins. It is not surprising, then, that lncRNAs have been associated with several physiological processes such as cellular differentiation, cell lineage choice, organogenesis, and tissue homeostasis (56). As a consequence, lncRNAs mutations or unregulated expression lead to the deregulation of said processes, and therefore to a number of pathologies.

For example, lncRNAs are implicated in cardiac development, but they are also recognized as key regulators of cardiovascular diseases. In fact, there are many lncRNAs implicated in cardiac functionality whose deregulation can promote cardiac hypertrophy and, in the long term, heart failure (57). Furthermore, many lncRNAs have been linked to angiogenesis and therefore to the pathogenesis of vascular disorders, that are in turn implicated in numerous pathologies (atherosclerosis, CAD, hypertension, vascular retinopathies, tumorigenesis) (58).

In the same way, lncRNAs can also be implicated in central nervous system (CNS) development or pathologies. While some, such as *Evf-2*, are involved in neuron differentiation and synaptic plasticity, many others were associated to the pathogenesis of famous neurodegenerative disorders (59). For example, the lncRNA *BACE1-AS* promotes Alzheimer is disease progression increasing the production of pro-amyloidogenic peptide Aβ42, of which brain senile plaques are composed. *BACE1-AS* is a natural antisense transcript of *BACE1*, which encodes the enzyme responsible for the production of Aβ42. It forms duplexes with *BACE1* mRNA, leading to its stabilization and increasing the production of both the enzyme and its product (59).

Cardiovascular and neurodegenerative diseases are only some of the conditions whose pathogenesis is associated or even induced by lncRNAs. Actually, we now know that lncRNAs are related to virtually all kinds of human pathologies, from infections (60) to preeclampsia (61), confirming their central role in human physiopathology.

### Long Non-Coding RNAs Role in Cancer

Cancer is included among the pathologies that are correlated to lncRNAs function and dysfunction. This is not surprising given the regulatory role that lncRNAs play in many processes that drive tumor progression. For example, lncRNAs are known to be involved in stemness, cell proliferation and angiogenesis (40, 58, 62), all of which can lead to the acquisition of malignant features, if deregulated. Furthermore, lncRNAs are fundamental epigenetic regulators directing the deposition of active or repressive marks on chromatin (6), and epigenetic alterations are universally acknowledged as determining factors in malignant transformation (63).

Furthermore, lncRNAs relevance in cancer is testified by the fact that they can regulate or be regulated by oncogenes and oncosuppressors. Therefore, it makes sense that lncRNAs could be part of perturbed pathways leading to tumorigenesis, tumor progression, and metastasis. The well-known oncogene P53 regulates the expression of a number of lncRNAs, that in some cases mutually counter-regulate it. For example, P53 represses *H19* expression, while the *H19*-derived miR-675 inhibits *P53* mRNA (64). There are also other oncogenes regulating lncRNAs, for example a well described pathway including *MYC* triggers the expression of several lncRNAs (65).

As a matter of fact, more and more lncRNAs are found deregulated in cancer, and in particular upregulated, suggesting a pro-tumorigenic role. In this respect, one of the best-known examples, *HOTAIR* is overexpressed in breast, hepatocellular, colorectal, pancreatic, lung and ovarian cancer, in which its upregulation correlates with tumor invasiveness and metastasis (66).

On the other hand, examples of lncRNAs downregulation associated with tumorigenesis are increasing every day, and high levels of expression of some lncRNAs are associated with a good prognosis, pointing toward their potential tumor-suppressor role. This is the case of *LincRNA-P21*, whose high levels correlate with progression-free and overall survival in diffuse large B cell lymphoma (67) and with enhanced sensitivity to radiation in colorectal cancer (68).

Another notable example of lncRNAs deregulated in cancer are those transcribed from ultraconserved regions (T-UCRs). T-UCRs expression pattern is remarkably different in healthy tissues and cancer, following an aberrant epigenetic regulation either through miRNAs, DNA methylation, or histone modifications (69). In some cases, their deregulation was also mechanistically linked to pro-tumoral events; for example, uc.338 promotes the cell cycle progression from phase G1 to S, boosting cell proliferation (70).

Interestingly, many cancer-associated SNPs that occur in non-coding regions are located in lncRNA genes, suggesting that not only the deregulation of their expression, but also their mutation could act in a pro-tumorigenic direction. For example, several risk-related SNPs lie within the lncRNA *HULC*, which is overexpressed in a number of cancers (71, 72), but also in the lncRNA *ANRIL* (73); while some polymorphisms of lncRNA H19 are correlated to bladder cancer (74).

Beyond these suggestive observations, the contribution of many lncRNAs to cancer development, evolution or metastasis is well established and even mechanistically explained. There are now multiple examples showing how the perturbation of the expression level or the structure of a lncRNA, due to copy number alteration, point mutation or other phenomena, impacts cellular processes and leads to tumoral phenotypes.

In the next chapter we will provide a few examples of the mechanisms through which lncRNAs can promote tumorigenesis in a case of interest: testicular cancer.

## Long Non-Coding RNAs Role in Testicular Cancer

Testicular tumors are the most common solid malignancy among young men aged 15–40 years, and although having a low incidence in the general population, their frequency is slowly, but steadily rising (75, 76). Their classification is based on their cellular origin: testicular germ-cell tumors (TGCTs) account for 95% of testicular cancers and can be divided into seminomas and non-seminomas (77). As the name suggest, TGCTs arise from the cancerogenic transformation of gonocytes whose differentiation into spermatocytes can be blocked at different stages: earlier for seminomas, later for non-seminomas, which can be further divided following their degree of differentiation (78).

Independently from its origin, testicular cancer is generally considered a tumor with a favorable prognosis. Thanks to a high responsiveness to cisplatin-based treatments even at metastatic stage, the 5 year-survival rate of testicular tumors approaches 95% (79, 80). Nevertheless, there are differences in the survival outcome of patients depending on the tumor type and the extent of disease, and in worst-case scenarios the 5 years progression free survival can drop to 41% (81).

As previously mentioned, testicular tumors are generally highly responsive to the standard treatment of combined orchiectomy and cisplatin-based chemotherapy. However, the current therapy presents some considerable problems. Cisplatin resistance develops in approximately 10–15% of cases, leading to tumor relapse after initial treatment or to refractory disease (82). Moreover, it is well-known how treatment-related complications, such as second malignant neoplasms, cardiovascular diseases, hypogonadism, infertility, and chronic cancer-related fatigue, represent major issues for young survivors (83).

Developing alternative therapeutics and granting an early detection of the tumor is crucial in order to limit the administration of cisplatin-based treatments and therefore improve the expectancy and the quality of life of the survivors, avoiding prolonged convalescence, rehabilitations, or oncologic sequelae. This can be mainly achieved through the discovering of new drug targets and biomarkers, which could be exploited in the therapeutic or diagnostic processes.

In this respect, lncRNAs are an emerging class of molecules that is gaining more and more interest for their potential clinical application in oncology. As we already illustrated, lncRNAs can participate in both tumorigenic or tumor-suppressing events and interact with known oncogenes and oncosuppressor in order to regulate cancer development, progression and even therapy resistance.

Therefore, lncRNAs represent a novel drug target whose expression could be modulated in tumoral cells through the use of targeted molecules: siRNAs and shRNAs to induce RNA interference, antisense oligonucleotides (ASOs) to trigger RNase-H-mediated degradation, or even small molecules which could specifically bind the lncRNA secondary structure. All of these strategies have either been proven effective *in vitro*, or have been experimented clinically on other ribonucleic molecules, such as miRNAs or mRNAs, so they could represent a concrete therapeutic option in the close future (84, 85). For example, the use of locked nucleic acid (LNA) gapmeR ASOs to induce the degradation of tumour-promoting lncRNA *MALAT1* was tested as a possible treatment for multiple myeloma (MM) with good results (86).

Furthermore, lncRNAs possess some features that would make them ideal biomarkers. As previously mentioned, they show a very tissue/cell-specific expression pattern, a high stability and they can be secreted in extracellular fluids, characteristics which would facilitate their detection in plasma or urine, eliminating the necessity of invasive procedures. The possibility of using lncRNAs as oncological biomarkers has not only been tested, but also turned into a reality with the FDA-approved test PROGENSA^®^ PCA3 assay by Gen-Probe Inc. for prostate cancer diagnosis, which is entirely based on the detection of lncRNA *PCA3* in patient urine (87–89).

**Figure 3:**
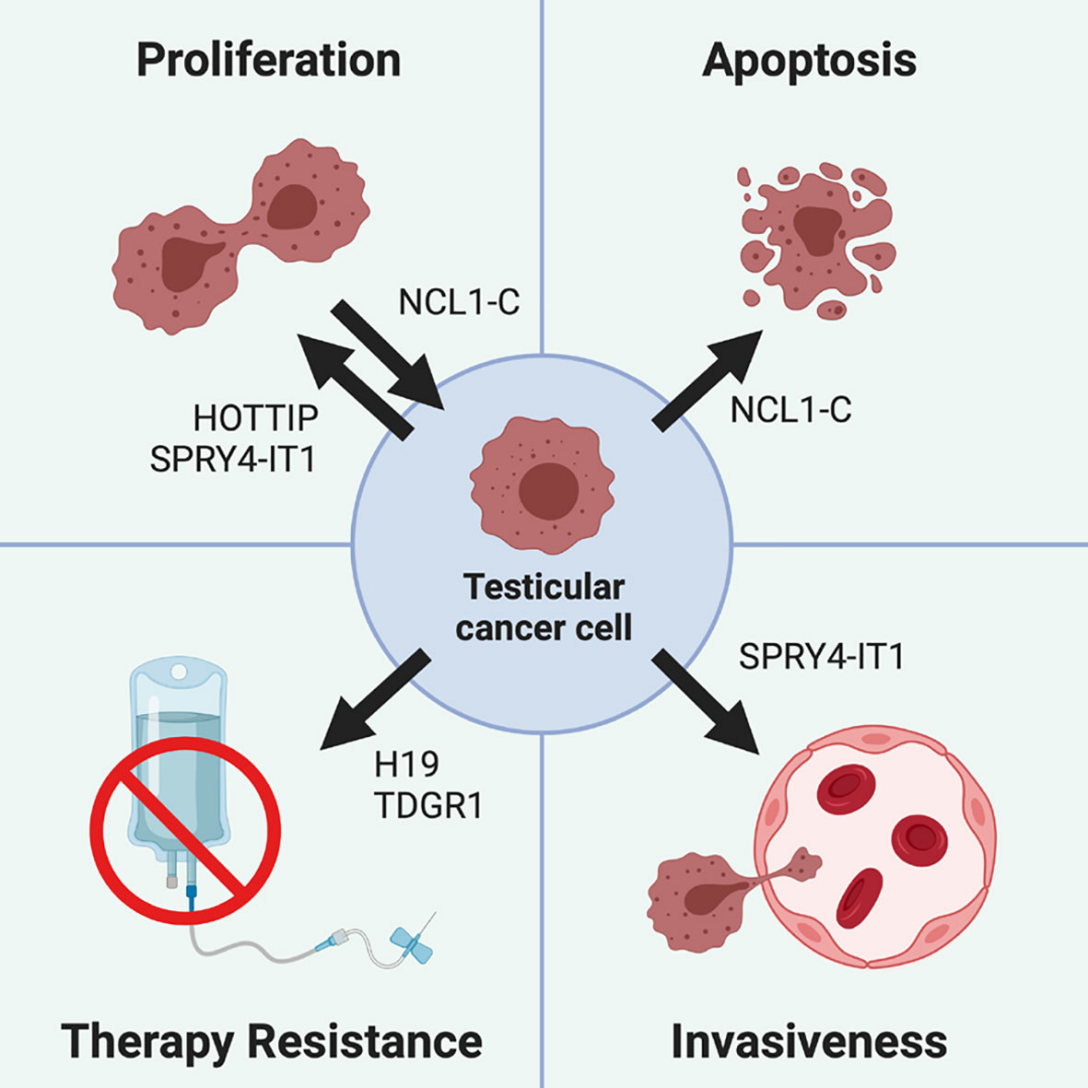
Role of different lncRNA in testicular cancer pathogenesis. Testicular cancer cells proliferation is promoted by lncRNA *HOTTIP* and *SPRY4-IT1*, while it is inhibited by *NCL1-C*, which also drives testicular cancer cells apoptosis. Cisplatin-based treatments resistance can be promoted by the *H19*/*TDRG1* pathway, since *H19* is able to upregulate *TDRG1*, which in turn triggers therapy resistance. Finally, testicular tumor invasiveness is promoted by *SPRY4-IT1*, which not only increases the cell proliferative rate, but also their motility and invasiveness.

Even though lncRNAs represent a very feasible alternative to traditional diagnostic and therapeutics tools for cancer management, their clinical application for testicular tumors pathogenesis is still largely mysterious. The relationship between some of these transcripts and testis neoplasia has been known literally for decades (90), however much remains to be discovered. In the following chapters we will provide an exhaustive description of all the lncRNA that have been associated to testicular cancer to our knowledge (**Table 1**, **Figure 3**).

**Table 1:** Overview of relevant long non-coding RNAs (lncRNAs) in testicular tumors pathogenesis.

Name	Alteration	Mechanism of action	Effect on tumorigenesis	Clinical studies	References
XIST	Expression in supernumerary X	Unknown	Unknown	Yes, as biomarker	(90–94)
H19	Loss of imprinting	Sponges miRNA‐106b‐5p causing TDRG1 upregulation	Pro-tumorigenic: promotes cisplatin resistance	No	(95–99)
SPRY4-IT	Overexpression	Unknown, possibly involving Akt pathway activation	Pro-tumorigenic: promotes cell growth, migration, and invasion	No	(100)
NLC1-C	Downregulation	Blocks miR-320a and miR-383 transcription through its binding to Nucleolin	Anti-tumorigenic: regulates cancer cell proliferation and triggers apoptosis	No	(101)
HOTTIP	Overexpression	Sponges miR‐128‐3p	Pro-tumorigenic: increases cancer cell proliferative rate	No	(102)

### X-Inactive Specific Transcript

In 1997 Looijenga and colleagues demonstrated how *XIST* expression is reactivated in testicular germ cell tumors (TGCT) following the acquisition of supernumerical X chromosomes. However, subsequent studies showed how the methylation pattern and the expression of X-linked genes in these cells were not consistent with a full inactivation of the supernumerical X, suggesting that *XIST* in TGCTs does not display its classical function of epigenetic silencer (91). An excess of X dosage in these tumors is justified by the presence of different X-linked oncogenes (91), however the biological meaning of *XIST* re-activation and its defective action in spite of its upregulation remains at this time unexplained.

Nevertheless, the presence of *XIST* expression, as well as epigenetic signatures typical of the inactive X chromosome, were proposed as biomarkers for testicular cancers. Typically, active X chromosomes present 5’ end methylation of *XIST* gene, while the allele on the inactive X is unmethylated. Thus male cells, which do not normally have an inactive X chromosome, are not expected to present an unmethylated *XIST* 5’ end. As just mentioned, the presence of supernumerary X chromosomes in TGCTs determine the presence of a partially inactivated X chromosome in which *XIST* is not transcribed, and whose 5’ is thus found unmethylated.

Different studies demonstrated not only that is possible to detect unmethylated *XIST* fragments in the plasma of some TGCT patients (92), but also that the overall *XIST* methylation levels are distinctly lower in TGCT patients that in healthy males (93). Recently, this epigenetic signature was also considered as a potential biomarker for testicular cancer detection and differential diagnosis, since *XIST* 5’ demethylation levels are significantly higher in seminomas than in non-seminomas or normal testis (94). Even though this study was performed in a large cohort of patients, and replicated in a second independent cohort, the analysis of unmethylated *XIST* fragments was only performed on testis biopsies and retrospectively.

It is apparent, then, that diagnostic tests based on *XIST* methylation status detection are still far from being a reality. Independent and prospective validation is required in order to consider *XIST* demethylation as a reliable biomarker for TGCT detections, and such tests should possibly be improved in order to allow a detection in plasma/urine, thus sparing the patients from invasive procedures.

### *Testis Developmental-Related Gene 1* and H19

Testis developmental related gene 1 (*TDRG1*) is a lncRNA that promotes the tumorigenesis and progression of a number of tumors, among which epithelial ovarian cancer, uterine cancers, gastric carcinoma, NSCLC, and osteosarcoma (103–111). Interestingly, *TDRG1* was first described as a protein-coding gene encoding for a 11kDa product expressed in mature testis (112). Such peptide is upregulated is seminoma, in which it is demonstrated to promote tumor growth, progression, and chemoresistance to cisplatin, a major challenge in seminoma treatment (113–116).

*TDRG1* peptide action in seminoma mainly relies on the activation of PI3K/Akt/mTOR oncogenic signaling pathway, since it is able to positively regulate the expression levels of p-PI3K, p-Akt, and p-mTOR, as well as to affect the translocation of nuclear p-Akt (113). Remarkably, lncRNA *TDRG1* was described to activate the same pathway in endometrial carcinoma and osteosarcoma (107, 111), thereby one could argue that PI3K/Akt/mTOR pathway activation in seminoma is also ascribable to this lncRNA.

Being only 100 nucleotides long, *TDRG1* proteic product is short enough to be considered a micropeptide, and thus it could simply be a product of a leaky translation of lncRNA *TDRG1*, and not that of an independent mRNA. In this scenario, the increase in the levels of *TDRG1* peptide would be reflective of that of the corresponding lncRNA and would not have a biological relevance.

However, these are only speculations, since the structure of *TDRG1* genomic locus has not been investigated enough to verify the presence of different transcriptional units. Moreover, the fact that *TDRG1* peptide could be translated from the lncRNA would not necessarily imply its lack of functionality. In fact, *TDRG1* peptide was also described to promote seminoma cisplatin chemoresistance inhibiting mitochondria-mediated apoptosis and promoting autophagy, mechanisms which have not been described for *TDRG1* lncRNA and which could support a functional relevance of the peptide (116, 117).

Independently of the nature of the molecule triggering cisplatin resistance in seminoma, *TDRG1* expression in this cancer is linked with that of a well-known lncRNA: *H19*. This lncRNA has long been known to be upregulated in testicular cancer, since its loss of imprinting is quite a common feature of TGCTs (95–97, 118).

In somatic cells, *H19* is paternally imprinted and maternally expressed, meaning that only the maternal allele of this gene is expressed, while the paternal allele is hypermethylated, and thus silenced. Biallelic expression of *H19* is physiologically found at early stages of embryogenesis, during which the parental imprinting signatures are transiently erased thanks to an epigenetic reprogramming. Therefore, *H19* loss of imprinting was initially thought to reflect the embryonal origin of TGCTs (98, 99).

However, in 2018 Wei and colleagues showed how *H19* upregulation could in fact directly promote testicular cancer pathogenesis. They found that the levels of *H19* and miRNA‐106b‐5p were inversely correlated, and that low levels of miRNA‐106b‐5p were associated with a higher *TDRG1* expression (117). Following bioinformatic considerations, and after an *in-vitro* validation of the model, the team proved that H19 acts as a miRNA sponge for miRNA‐106b‐5p, thereby impairing the miRNA activity on its target gene, *TDRG1* (117). In this way, *H19* would directly lead to *TDRG1* upregulation, leading to seminoma cisplatin resistance.

### SPRY4 Intronic Transcript 1

*SPRY4* intronic transcript 1 (*SPRY4-IT1*) is a lncRNA derived by the second intron of *SPRY4*, a protein-coding gene encoding for a regulator of receptor tyrosine kinases (RTKs) (119). Unsurprisingly, alterations in *SPRY4* expression levels have been associated to a number of cancers, given that it is able to modulate both MAPK/ERK and PI3K/Akt pro-tumorigenic signaling pathways (120).

*SPRY4-IT1* was first detected in 2011 in melanoma (119), and since then an increasingly expanding literature has developed regarding its role in oncogenesis. In general, *SPRY4-IT* seems to act in a pro-tumorigenic sense, being associated to the development and progression of several tumors, among which breast cancer (121, 122), osteosarcoma (123, 124), and colorectal cancer (125, 126). However, there are some cases in which *SPRY4-IT4* has been reported to act as a tumor suppressor, in particular in non-small cell lung cancer (127, 128).

Das and colleagues reported that both *SPRY4* and *SPRY4-IT1* are upregulated in TGCT, and that they may act as oncogenes promoting the activation of the PI3K/Akt signaling pathway (100). In fact, *SPRY4* and *SPRY4-IT1* knockdown *in vitro* resulted in decreased tumor growth, migration, and invasion, also leading to a significant reduction in Akt phosphorylation (100).

While *SPRY4* action on PI3K/Akt pathway was not surprising, *SPRY4-IT1* role in this context was unexpected and its mechanism of action is still unknown. Different studies reported a transcriptional and functional independence between *SPRY4* and *SPRY4-IT1* (120, 129), so the function of the latter should not be sought in their interaction. Nevertheless, there is another cancer that could give insights on *SPRY4-IT1* action in the regulation of RTK-mediated signaling pathways.

Melanoma is not only the first tumor in which *SPRY4-IT1* was detected, but also one of the few cancers expressing both *SPRY4* and *SPRY4-IT1* at high levels (120). Similarly to what happens in TGCTs, these genes are upregulated in melanoma in regards to normal melanocytes, and they act as oncogenes in different fashion (120). In particular, *SPRY4-IT1* was reported to act as a miRNA sponge to regulate the phosphorylation of various members of the MAPK oncogenic signaling pathway, thus increasing cancer cell proliferation and motility (129).

This same mechanism could be shared by *SPRY4-IT1* in TGCTs, which could be able to control Akt phosphorylation levels sequestering a miRNA through base-pairing interaction. *SPRY4-IT1* has been seen to sponge at least three different miRNAs in several types of cancers (122–124, 130–134), so it is possible that emerging interactions with other miRNAs could mechanistically explain *SPRY4-IT1* role in TGCTs. It is also important to notice, though, that *SPRY4-IT1* is predicted to contain several long hairpins in its secondary structure (120), which could serve as a platform for its interaction with proteins. Therefore a direct interaction with Akt or one of its regulators is not to be dismissed.

### Narcolepsy Candidate-Region 1 C

Narcolepsy candidate-region 1 C (*NLC1-C*) is a lncRNA first identified as a potential candidate gene for narcolepsy resistance (135). It is highly expressed in human sperm and brain, particularly in the white matter of the frontal lobe (135, 136).

*NLC1-C* expression in spermatogonia and early spermatocytes is reflective of its role in the early stages of spermatogenesis (101). The formation of mature sperms is a complicated and highly coordinated process which requires not only an initial proliferation of spermatogonia, but also a loss of germ cells during spermatocytes meiosis and spermatids differentiation (137). Under this perspective, *NLC1-C* role is that of controlling the proliferation of germ cells to ensure a successful spermatogenesis, thus preserving male fertility (101).

From the molecular point of view, *NLC1-C* action is based on its interaction with nucleolin, a nucleolar protein involved in several RNA regulatory mechanisms, including transcription, ribosome assembly, mRNA stability and translation, and miRNA processing (138, 139). Together, *NLC1-C* and nucleolin bind the promoters of miR-320a and miR-383, hampering their transcription and regulating the proliferative activity of germ cells (101). In return, miR-320a and miR-383 regulate *NLC1-C* cytoplasmic levels though RNA-interference, creating a well-balanced network (101).

*NLC1-C* was found downregulated both in the cells of patients affected by uniform testicular maturation arrest (MA), a clinical characteristic associated with male infertility, and in testicular embryonal carcinoma cells (135). Interestingly, *NLC1-C* downregulation was associated with an altered intracellular localization, with an overexpression of the lncRNA in the nucleus compared to the cytoplasm (101). This alteration determines an increased binding of *NLC1-C* to nucleolin and therefore a hyperactive proliferation of germ cells *via* the downregulation of miR-320a and miR-383, ultimately leading to MA (101).

The same process is responsible for *NLC1-C* pro-tumorigenic role in testicular cancer. *NLC1-C* expression levels influence the survival of testicular embryonal carcinoma cells: while its downregulation promotes their proliferation, *NLC1-C* upregulation not only ends up in a reduction of the proliferative rate, but in an increase of apoptosis (101). This phenomenon is again mediated by *NLC1-C*/nucleolin regulation of miR-320a and miR-383, which can influence the levels of several molecules involved in the cell cycle progression and apoptosis (p21, activated caspase 3,8,9 and PARP) (101).

The fact that *NLC1-C* is downregulated in both MA and testicular embryonal carcinoma is consistent with the observation that infertile men have a higher risk to develop testicular cancer (140–142), and it is particularly interesting since it offers a molecular link between the two pathologies. Furthermore, this study underlines a new pathway that could be used as possible drug target in testicular cancer treatment, or for its prevention in susceptible individuals (as infertile males).

It is interesting to notice that miR-320a and miR-383 onco-suppressive role have already been described in a number a tumor, and in some contexts has also been mechanistically characterized. Therefore, other oncological settings could provide more information regarding miR-320a and miR-383 targets and function in testicular cancers.

For instance, miR-320a downregulation has been linked to chemoresistance in a number of cancers, including gastric cancer, in which its interaction with metallopeptidase ADAM10 seem to promote cisplatin sensitivity (143). If such a function was confirmed in testicular cancer, it could be of great interest for a potential use in cisplatin resistant tumors, which as we said represent particularly challenging cases.

On the other hand, miR-383 is not only correlated with cell cycle progression and apoptosis, but it was also described to regulate cancer progression, migration and invasion in different tumors, such as gastric cancer, cholangiocarcinoma and hepatocellular cancer (144–146). Under this perspective miR-383 downregulation could be useful not only as a predictive biomarker, but also to determine the prognosis of cancer patients.

### HOXA Distal Transcript Antisense RNA

*HOTTIP* is a lncRNA encoded by a genomic region in the 5′ tip of the *HOXA* locus. Initially its function was only linked to the transcriptional regulation of this locus during embryogenesis, since this lncRNA can recruit WDR5/MILL complex thus leading to the expression of the *HOXA* genes (147). However, in the past few years *HOTTIP* was discovered to be have a pivotal role in human tumorigenesis, being expressed in nearly all kinds of human cancers (148).

The oncogenic role of *HOTTIP* is not solely mediated by *WDR5* action, but also by the interaction with several different chromatin regulators, oncogenic pathways, and miRNAs depending on the cancerous setting in which is involved (102). In testicular embryonal carcinoma, *HOTTIP* was described to promote cell proliferation *via* the competitive binding to miR‐128‐3p (102).

In fact, *HOTTIP* was found upregulated in testicular embryonal carcinoma cells compared to the control, and its knock-out in those cells resulted in a decrease of the proliferative rate (102). Bioinformatic considerations based on *HOTTIP* structure, later validated *in vitro*, proved that its effect on cell proliferation was mediated by miR‐128‐3p, that is sponged by *HOTTIP* (148). This miRNA has a well-established anti-proliferative action (149–151), therefore its inhibition by *HOTTIP* is consistent with the phenotype detected in testicular embryonal carcinoma cells.

Furthermore, *HOTTIP*/miR‐128‐3p pathway was found to positively regulate *HOXA13* expression, which is quite a canonical downstream effector of *HOTTIP* in cancerogenic settings (102, 148). In fact, overexpression of *HOXA13* in response to *HOTTIP* oncogenic action was described in small cell lung cancer, gastric cancer, pancreatic, hepatocellular and esophageal squamous cell carcinoma, and in some of these setting its expression alteration is triggered by the inhibition of a miRNA (148).

If its correlation with testicular cancer were to be further investigated, *HOTTIP* could represent an excellent biomarker. In fact, *HOTTIP* not only has been tested as a potential biomarker in several different tumors, and notably as a non-invasive biomarker in colorectal cancer (152), but several of its SNPs inside of it are associated to cancer susceptibility, prognosis, or therapy response (148). The possibility of having such biomarkers for testicular cancer would determine a better allocation of therapeutic resources and would spare good-prognosis patients from toxic treatments.

## Discussion and Conclusive Remarks

LncRNAs are now recognized as fundamental regulators of several physiological and pathological processes. They take part in a number of cellular events, regulating gene expression, nuclear organization, as well as the activity of several signaling pathways. In particular, their role in cancer development and progression is gaining more and more attention as their involvement is discovered in a number of tumor-promoting or suppressing events.

In fact, copy number alterations or point mutations affecting lncRNAs, as well as their aberrant expression, can promote tumorigenesis, leading to their identification as an emerging class of oncogenes/oncosuppressors. Studying further their mechanisms of action in cancerous settings will not only allow us to better understand cancerogenic networks, but also to detect or disrupt them by targeting/exploiting the action of lncRNAs.

The use of lncRNAs for cancer detection and treatment is particularly interesting for cancers in which conventional therapeutics and biomarkers are scarcely effective or cause major side effects. That is the case of metastatic testicular cancer, in which lncRNAs could limit the administration of highly toxic cisplatin-based chemotherapy, currently the main therapeutic option to treat this malignancy.

The prospect of using lncRNAs as drug targets or therapeutics is still under development, and even though very promising also presents some difficulties. On one hand some of the approaches that are being explored to target lncRNAs have already been tested on other molecules and proven successful in treating cancer. On the other hand, the poor conservation of lncRNAs among different species could represent a challenge for the development of therapeutic strategies in animal models, that could lack the orthologue of the lncRNA of interest.

Nevertheless, lncRNAs have already been employed in cancer diagnostics. Their tumor/tissue specificity, as well as the possibility of dosing them in body fluids make them ideal biomarkers for neoplastic pathologies. Moreover, since their aberrant expression may either indicate the presence, the stage or the treatment-resistance of the tumor, they can have a broad range of applications at each step of the malignancy management.

In the case of testicular cancer, few lncRNAs have been linked to its pathogenesis (153, 154), and even though no clinical studies are available for many of them, these lncRNA may represent promising drug targets or biomarkers.

Both *SPRY4-IT1* and *HOTTIP* were shown to increase testicular cancer proliferation (100, 102), so their inhibition or downregulation could lead to a decrease of tumor aggressiveness and size. This would make adjuvant chemotherapy dispensable for localized masses, which could be more easily removed *via* surgical excision.

Conversely, *NLC1-C* is downregulated in testicular cancer, and its reactivation lead to a decrease in proliferation and an increase in apoptosis of cancer cells (101). Therefore, forcing *NLC1-C* re-expression in testicular tumors could prove to be a feasible therapeutic alternative to cisplatin-based chemotherapy.

Targeting other lncRNAs could be useful to revert resistance against conventional therapeutics, and thus could be used in association, instead of in substitution, with cisplatin-based chemotherapy. That is the case of *H19*, which drives cisplatin resistance in testicular tumors (117), and whose inhibition could restore cancer cells sensitivity to these chemotherapeutics.

Finally, lncRNAs could be used as diagnostic, prognostic, or predictive biomarkers for testicular cancer: the methylation levels of 5’ *XIST* fragments could help detect testicular cancer, the presence of peculiar polymorphism in *HOTTIP* could determine its prognosis, the upregulation of *SPRY4-IT1* its metastatic potential, and the upregulation of *H19* the responsiveness to cisplatin-based treatments.

To conclude, lncRNAs surely deserve the attention of the scientific community, especially when it comes to cancer research, and hopefully studying them will give us a new perspective on the mechanisms behind neoplastic pathologies and allow us to develop new strategies for their detection and treatment.

## Author Contributions

CB and MB conceived the review. VV and BC were responsible for its critical revision. All authors contributed to the article and approved the submitted version.

## Conflict of Interest

The authors declare that the research was conducted in the absence of any commercial or financial relationships that could be construed as a potential conflict of interest.
